# Bedtime Routines Intervention for Children (BRIC) using an automated text messaging system for behaviour change: study protocol for an early phase study

**DOI:** 10.1186/s40814-020-0562-y

**Published:** 2020-02-06

**Authors:** George Kitsaras, Julia Allan, Iain A. Pretty

**Affiliations:** 1 Dental Health Unit, Division of Dentistry, The University of Manchester, Manchester, UK; 2 Institute of Applied Health Sciences, University of Aberdeen, Aberdeen, UK

**Keywords:** Bedtime routines, Child, Development, Wellbeing, Digital health technologies, Behaviour change

## Abstract

**Background:**

This work concerns the activities in the last hour before bed for young children born to first-time parents, so called bedtime routines (BTR). These activities include amongst others tooth brushing, reading a book, having a bath and avoiding food and drinks before bed. Having a set bedtime at a suitable hour is also very important. Establishing good bedtime routines has been shown to be really important for a number of health, wellbeing, development and social outcomes. Currently, there is no evidence-based bedtime routine intervention for first-time parents using a novel design (i.e. text messages). Existing research has highlighted the importance of bedtime routines and the lack of appropriate mechanisms in place for parents who sought support.

**Methods:**

The proposed study includes 2 work packages. Work package 1 focuses on the development of the intervention through a combination of qualitative work (1:1 interviews with parents on barriers and facilitators on bedtime routines using the Theoretical Domains Framework) and an expert group of key stakeholders. Work package 2 involves a small-scale (*n* = 50) feasibility and effectiveness study to examine proof of concept with first-time parents using text messages to communicate the intervention. Quantitative information relating to uptake, engagement, retention and effectiveness of the intervention as well as qualitative information (focus groups with parents who took part in the study) will be collected. Overall, the effectiveness of the intervention will be assessed through the APEASE criteria (acceptability, practicability, effectiveness, affordability, safety, equity).

**Discussion:**

This study can provide initial yet important support for further exploration in the field of bedtime routines in more complicated family structures (parents with more than 1 child, separated families etc.). Also, the implementation of a novel study design (i.e. text messages) could lead to considerable cost savings while maintaining high retention, uptake and engagement from the participants. Should the intervention meet the APEASE criteria, a more comprehensive intervention on bedtime routines for first-time parents will be explored in a more robust (RCT and longitudinal) approach.

**Trials registration:**

Due to the nature of the study, no trial registration is currently in place.

## Background

Bedtime routines are the single most frequent and recurrent activity for families with young children [1, 2]. Bedtime routines are established gradually around the first year of a new-born’s life, and by the end of infancy (year 2), most families have well-established routines [1]. During bedtime routines, a series of repeated behaviours (i.e. tooth brushing, book reading, going to bed at a consistent time, dietary habits/avoiding sugary snacks etc.) take place. These behaviours can have implications for oral hygiene and dental health [3], quality of sleep [4], school performance and school readiness [5], psychosocial development [6], and behavioural and cognitive development [6] as well as parental socioemotional wellbeing [6] and overall family functioning [1–6]. Bedtime routines therefore are not just relevant to a single public health issue. They cover a combination of health, developmental, social and behaviours with short- and long-term implications for young children, their parents, public finances and the health service. An intervention targeting first-time parents of young children is especially important given the crucial link between early life experiences, later achievement and wellbeing for children as well as the importance of assisting and supporting young parents highlighted by other publicly funded projects like baby boxes, Dental Checks by 1 and Head Start in the USA [1, 5, 7]. These interventions apart from their focus on children provided parents, as the primary caretakers for young children, with important skills, training, information and education resources to allow for greater effectiveness [6].

The lack of good oral hygiene behaviours around bedtime and consumption of snacks/drinks in the hour before bed increases the likelihood of dental caries [7]. The negative impact of dental caries in young children includes chewing difficulties, sleeping difficulties, changes in behaviour (e.g. irritability), adverse psychological development (such as low self-esteem) and loss of school days with impact on school performance [8, 9]. Also, if left untreated, dental caries can lead to extractions under general anaesthetic with further implications for children’s psychosocial wellbeing with increased pain, need for hospitalisation, increased anxiety for parents as well as public finances and public healthcare pressures [9]. In just one financial year, the NHS has spent almost £60 million on preventable tooth extractions in young children [10], and it is the commonest reason for children to be admitted to hospital. In general, dietary habits around bedtime have shown important associations with obesity rates with snacking before bed linked to higher BMI, while those with significant dental decay are often undernourished [11]. Book reading with children as part of the bedtime routine can promote child literacy, improve school performance and enhance school readiness in young children with subsequent possible implications in later achievement and attainment [12]. School readiness at age five is considered key for successful grades at school, reducing high school dropout and higher earnings in adulthood [13]. Finally, having a consistent, appropriate time children go to bed could aid in achieving adequate hours of sleep with important consequences for psychological wellbeing, physical health, family functioning, school achievement and optimal brain development [4].

Despite growing evidence on their importance, bedtime routines remain underresearched [5]. Existing attempts in changing bedtime routines in families with young children have either utilised non-automated, resource intensive approaches [12] or focused on selective bedtime routines behaviours rather than the entirety of the routine and its different components [6]. Despite some shortcoming, existing interventions have showcased the potential of changing and maintaining optimal behaviours around bedtime routine-related activities. For example, an intervention on literacy skills development linked to book reading before bed showed increased and sustained changes for those targeted behaviours (book reading/book sharing before bed) for the intervention group when compared to the control [12].

Nevertheless, issues with past studies and existing interventions create a unique opportunity for developing a novel, automated, text message-based intervention that addresses all aspects of bedtime routines for first-time parents. Despite preliminary work from this research team, an early phase study is important for gaining a better understanding of bedtime routines in first-time parents, an understanding that will allow for the development of an evidence-based intervention. This research team has completed one of the few bedtime routine-related studies in the UK with families with young children with promising results regarding the importance of bedtime routines and the incorporation of text message-based applications for families with young children [14, 15]. However, the creation of such a novel intervention requires preliminary technical development and testing that can be achieved through an early stage study. In effect, the proposed early phase study will act as a bridge between existing evidence on bedtime routines and the transformation of that knowledge into a practical, evidence-based intervention that can help first-time parents.

Text messages will be at the heart of the proposed intervention. Text messages are going to be used given the high percentage of people who own a working mobile device (93% in the UK) [16], the successful use of text message interventions targeting other health-related behaviours [17], their popularity as a means of communication in general and amongst ethnic minorities and deprived communities as well as their overall low cost per participant [17, 18]. Moreover, text messages as an observational data collection tool were successfully utilised by this research team in a study of bedtime routines with families with young children with extremely positive feedback on the lack of intrusiveness, ease of use and interface [15]. Finally, avoidance of app-based or computer-based systems or approaches is considered beneficial in reducing harm through limited screen time exposure for parents.

### Objective

The objective of this public health early phase study is to develop and test an intervention to support the adoption and maintenance of optimal bed time routines for first time parents that will lead to measurable improvements in health, development and wellbeing for young children. The intervention will utilise real-time, responsive text messages that are co-designed and developed with parents and experts. Proof of concept will be assessed through the APEASE criteria following a feasibility and effectiveness study.

## Methods/design

### Study design overview

**Fig. 1:**
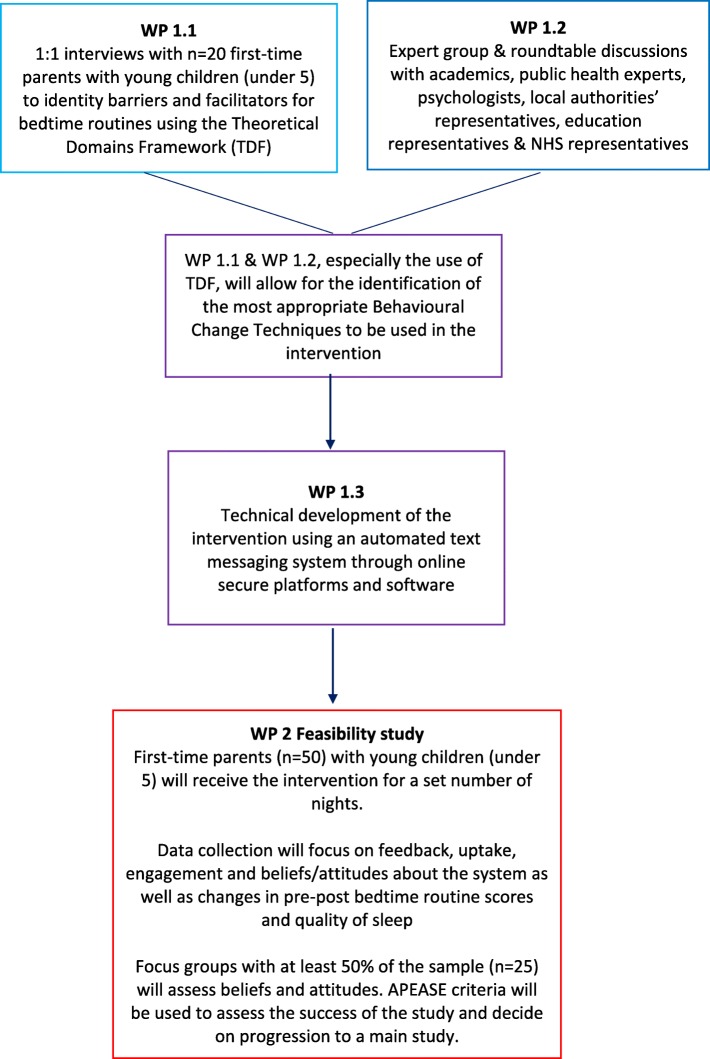
Flowchart of proposed early phase study. Flowchart describing each work package (WP) of the proposed early phase study including details on each WP

The early phase study will include 2 work packages (WP) over an 18-month period. User and stakeholder engagement will be in the core of each WP. Figure 1 presents an overview of the study and its individual working packages.

WP 1 (months 1–9) will focus on defining and developing the intervention. WP 1 will consist of 3 sub-packages: (WP 1.1) will identity barriers and facilitators for establishing and maintaining good bedtime routines; (WP 1.2) will include an expert group of key stakeholders, academics and researchers; and finally (WP 1.3) will involve the technical development of the system used to deliver the intervention. WP 2 (months 10–18) will include an uncontrolled quantitative and qualitative feasibility and effectiveness study using the text message-based bedtime routine intervention.

### Eligibility

For WP 1.1 (qualitative work) and WP 2 (feasibility and effectiveness study), only first-time parents with young children (between 1–3 years old) will be considered for the study. Both parents will need to be first-time parents. Single parent families will be included as well as parents from academic/non-academic backgrounds. No age restrictions apply to participants. Exclusion criteria will include inability to comprehend English, no access to working mobile phone and not being a first-time parent.

### Participants

For WP 1.1 (qualitative work), it is expected that a total of 20 first-time parents will take place. Data saturation will be monitored throughout data collection, and once saturation has been achieved, data collection will stop. Data saturation estimates as based on previous work by the research team on bedtime routines barriers and facilitators for parents. The sample in WP 2 will consist of 50 first-time parents with young children. For the focus groups, at the end of WP 2, it is expected that at least 50% of participants who received the intervention (*n* = 25) will participate in semi-structured focus groups to provide feedback and their insight on the intervention they received. Convenience sampling, due to the nature of this study as a feasibility and effectiveness study, will be used for all working packages.

### Recruitment

For WP 1.1 (qualitative work) and WP 2 (feasibility and effectiveness study), recruitment will take place in non-healthcare settings with priority given at Sure Start Centres, nurseries and staff of the University of Manchester. Recruitment will be contacted directly by the research team who will conduct in-person visits at these locations. Full explanation of the study’s objectives and process will be provided to participants alongside a Participant Information Sheet. Consent will be obtained in person during recruitment.

### Intervention

The final intervention, to be examined at the feasibility and effectiveness study (WP 2), will include text messages around bedtime for first-time parents with young children (ages 1–3) designed to prompt, alter, reinforce and maintain optimal bedtime routines that achieve all necessary components (i.e. tooth brushing, reading a book etc.). The final intervention using text messages will build on existing cross-sectional work by this research team on bedtime routines in families [15].

Text messages will include evidence-based Behaviour Change Techniques (BCTs [19];) identified from the early phase study. BCTs are the ‘active ingredients’ within an intervention designed to produce a change in behaviour. Appropriate BCTs will be selected using the Behavioural Change Wheel (BCW [14];), a theory-based framework designed to guide intervention development in a structured, systematic and evidence-based way. The intervention will be aimed at first-time parents with young children from all sociodemographic/ethnic backgrounds. Text messages will be kept free for all participating families to ensure equal chances for participation. To achieve the expected outcomes, the intervention will be delivered for set period of time with the actual duration to be specified during the early phase study. Longer duration will allow for the necessary observations in terms of behaviour change, behaviour maintenance and impact on core outcomes. Each night, parents will be given the opportunity to opt out from the study using an easy opt-out clause (texting LEAVE). The exact content of the messages will be decided based on the results of the qualitative work (WP 1.1) since all content will need to be linked to appropriate BCTs based on the mapping of barriers and facilitators into the BCW.

### Data collection

For both WPs, demographic information will be collected through a quick demographics form at the point of recruitment.

For WP 1.1 (qualitative work), semi-structured interviews will be used; the interview schedule will be developed using the Theoretical Domains Framework (TDF, [20]), a framework which summarises 84 possible determinants of behaviour into 14 overarching ‘theoretical domains’. Structuring the interviews around the TDF domains will ensure comprehensive exploration of all possible determinants of suboptimal bedtime routines, therefore helping to identify the best targets for change in our subsequent intervention. Interviews will be conducted either in person or via telephone to minimise disruption and allow for a better participant experience. Interviews are expected, based on previous experience, to last around 30–45 min. A guide on the qualitative interview schedule can be found in Additional file 1.

Pre- and post-intervention scores on quality of bedtime routines and children’s quality of sleep will be collected using an adapted text message-based assessment of bedtime routines (Additional file 2) and the Child Sleep Habit Questionnaire [21]. The child sleep questionnaire will be completed by parents, and it will include questions on sleep habits for their children. This instrument contains a total of 22 questions on a 5-point Likert scale (always, usually, sometimes, rarely and never). Additional file 3 contains a copy of the questionnaire. The bedtime routine questionnaire is an instrument developed by the research team during their previous work in the area, and it reflects a dynamic approach in assessing bedtime routines as they happen [15]. To ensure accuracy, post-intervention measurements will take place near the end of the intervention and preferably within a week. However, some measurements, like the bedtime routine questionnaire, due to its nature as a text message-adapted assessment, will be sent to participants immediately after their final intervention text message.

Beliefs and attitudes towards the system will be examined by the focus groups at the end of the feasibility and effectiveness study (WP2). The focus group moderator will ensure that all participants are given the necessary space to express their views about the intervention with a special emphasis on their feedback and suggestions for improvement. Focus groups will include semi-structured questions on their experience of the intervention and their beliefs and attitudes as well as their recommendations for future changes in the system. Focus groups will be held at locations convenient to the sample. Focus groups are expected to last 1 h. A guide on the topics and issues to be covered at the suggested focus groups can be found in Additional file 4.

### Outcome measures

Expected outcomes for the intervention include short-term/process outcomes such as (a) improvement in bedtime routines for first-time parents including achieving and maintaining all important elements of an optimal routine (tooth brushing, book reading, avoidance of sugary snacks etc.), (b) improvement on quality of sleep for young children through a better routine, (c) less behavioural difficulties around bedtime (e.g. tantrums, resistance), (d) better parent-child interactions and (e) improved parent and children’s socioemotional state. WP 2 (feasibility and effectiveness study) will evaluate the intervention using the APEASE criteria ((a) acceptability, (b) practicability, (c) effectiveness, (d) affordability, (e) safety and (f) equity). APEASE criteria will be assessed through insight data regarding retention rates, response rates, user engagement, number/type of problems encountered, cost per participant, user feedback, preliminary changes in a bedtime routine score pre- and post-intervention, and user beliefs and attitudes towards the system. Table 1 presents an overview of each APEASE criterion and the way in which data collection and quantitative analysis will address each one of them.

**Table 1:** Addressing APEASE criteria

Acceptability and practicability	Effectiveness	Affordability	Safety	Equity
Retention and response rates (user engagement) Focus groups feedback	Pre/post-bedtime routine scores and quality of sleep	Focus groups feedback	Incidents/data security report	Cost per participant, retention and uptake

### Analyses

#### Qualitative analysis

For WP 1.1 (qualitative work), data analysis will follow a deductive approach where each response will be mapped into TDF domains with identified domains subsequently linked to the Behaviour Change Wheel in order to inform the selection of appropriate Behaviour Change Techniques. This approach, specific to the TDF, mirrors the elements of a thematic analysis for qualitative data, since themes are identified and then linked to specific domains within the framework. Overarching themes and key barriers and facilitators affecting the targeting behaviour will also be captured. The use of the TDF in the interviews will lead to a holistic understanding of the targeted behaviour in terms of the BCW/COM-B models. For the focus groups (WP 2), data analysis will follow a thematic approach where key and overarching themes alongside frequency counts of responses will be collected.

#### Quantitative analysis

Due to the nature of the study, quantitative data analysis will focus primarily on process outcomes including retention rates, response rates, user engagement, number/type of problems encountered, cost per participant, user feedback, preliminary changes in a bedtime routine score pre- and post-intervention, and user beliefs and attitudes towards the system. These analyses will be performed through a series of descriptive and frequency statistics as well as through a cost analysis to determine cost per participant. For the changes in bedtime routine scores, pre/post-intervention bedtime routine scores will be assessed using a bedtime routine score per participant as in [15]. That will allow for paired sample mean comparisons to determine significant changes.

### Expert group

An expert group will be chaired by Prof Michael P. Kelly (University of Cambridge) former director of Public Health for National Centre for Health and Care Excellence (NICE). The expert group will include key stakeholders from academia, research, clinical practice (mainly public health), educators, local authorities, the NHS and parent groups. Key stakeholder presence is vital for (a) gathering their expert opinion and feedback on the proposed intervention especially with regard to its delivery mechanisms and targeted outcomes and (b) to examine, especially with local authority and NHS representatives, possible routes for future funding and implementation of the proposed intervention. The expert group will be hosted in Manchester, UK.

### Technical and ethical considerations

The technical development and implementation of the intervention (WP 1.2) will include the use of SafeMessage. SafeMessage is a secure, online software platform for designing, developing, sending, receiving and managing research data through the use of text messages and text surveys. SafeMessage utilises UK-based secure servers hosted on the NHS (National Health Service) HSCN (Health and Social Care) network that meet all government requirements for data protection including the General Data Protection Regulation (GDPR). For the purpose of this project, SafeMessage will be the primary route of communication and intervention delivery for first-time parents. Parents will be logged into the system using their first name and personal mobile number, and they will pre-determine the time they want to receive the intervention based on what time they start with their bedtime routine. All information regarding participant data will be safely stored in SafeMessage secure servers, and they will be cleared at the end of the study.

Ethical considerations include working with first-time parents and their children as part of this project, while technical considerations include data and information management through the use of the software and the automated text-messaging system. Regarding the former, proposed activities have limited risk of causing harm to participants or their children. There will be no invasive processes associated with the early phase study. Children will not be actively involved in the early phase study, since their parents will be the ones receiving the text message behaviour change intervention. All aspects of the study will be designed in a way that minimises intrusiveness and keeps overall time requirements and commitment to a minimum. Also, the use of text messages instead of app-based or online approaches minimises risk of extensive screen time exposure. Participants will be made aware of study requirements and time commitment during recruitment, and informed consent will be sought from all participants at the time of recruitment. There will be an easy and automated process of opting out of the study at a time should they require to do so. All information relating to the study will be managed through the use of unidentifiable participants IDs. IDs will be issued during recruitment and used throughout the rest of the study. Hard copies of documents relating to the study (i.e. consent forms) will be electronically coded in password-protected, secure files and original hard copies will be stored separately, securely stored in locked cabinets at the University.

## Discussion

Making sure that every child is given an equal chance in life is a key priority for governments and policy makers around the globe (i.e. Head Start in the US, baby boxes in Nordic countries and Scotland etc.) [22]. Moreover, prominent expert groups, including the Lancet Early Childhood Steering Committee, have raised the importance of investing in the health and wellbeing of young children and first-time parents with special emphasis on recurrent, nurturing behaviours [23]. Bedtime routines are the commonest recurrent, nurturing family activity with important implications for later life, and first-time parents are particularly vulnerable to problematic routines [1]. A bedtime routine intervention for first-time parents has the potential to provide all of the necessary tools to achieve optimal routines with wide ranging short- and long-term benefits. Optimal bedtime routines include multiple preventive, beneficial health and social activities such as tooth brushing, good dietary habits around bedtime, book reading, consistent and appropriate bed times and positive child-parent interaction. Through optimal routines, children could potentially lead more fulfilling lives as adults with lower incidents of health, psychological and behavioural issues (e.g. dental caries and conditions, higher school readiness, better sleep hygiene etc.). These improvements can also lead to wider positive public finance and health service consequences through reduced need for treatment for dental caries, reduced hospitalisations, better school attainment and fewer school dropouts. Therefore, focusing on developing, establishing and maintaining optimal bedtime routines is an important step in ensuring the best and equal chances in life for all children.

### Dissemination plan

The proposed dissemination plan for this early phase study attempts to capture the dynamic and multi-faceted nature of bedtime routines and the diverse audiences who will be interested in the study results. The dissemination plan aims to (a) raise awareness around bedtime routines and their importance for child development-wellbeing; (b) improve understanding of the development, maintenance and change of bedtime routines; (c) create a connection between the research outputs, the research community and the wider public; and (d) win trust and secure advocacy regarding future research, development and funding of interventions around bedtime routines as well as utilisation of such interventions from users (i.e. parents).

To achieve these goals, at the end of the early phase study, it is proposed that a peer-reviewed article will be published that will summarise key findings of the feasibility and effectiveness study and also present the technical details relating to the development of the text messaging system. That publication will be in an open access journal allowing easy access for researchers, academics and the wider public. Moreover, all participants in both work packages and experts involved in the early phase feasibility and effectiveness study will be offered updates on the progress of the study including a summary of key findings at the end of the study. Additionally, special interest groups and key stakeholders including charities (e.g. ‘Book Trust’ due to their campaign for bedtime reading) and national organisations and bodies (e.g. Public Health England due to their Baby Teeth Do Matter and Dental Checks by 1 campaigns) as well as other groups (e.g. Centre for Behaviour Change and the Digital Health Hub at UCL due to their particular interest in behaviour change and digital behaviour change interventions) will be informed of the outcomes of this study through newsletters and direct communications.

Also, an informative, short video presentation summarising the early phase feasibility and effectiveness study and its key findings will be created and shared on social media (especially Twitter and Facebook) and online platforms (i.e. YouTube etc.) to raise awareness and reach a different demographic (young adults and parents) who might be more inclined to access, visit and view such social media platforms. Sharing and promotion of that video communication can be achieved through the official social media accounts of the University of Manchester and the University of Aberdeen. Finally, MRC itself will be a key partner in further communicating and disseminating the outcomes of this early phase study through articles sent to the MRC’s blog and updates sent to the press office at MRC.

### Beneficiaries

In general, bedtime routines remain a niche area of research with only a few studies focusing on them. Research on bedtime routines has focused primarily on their link to quality of sleep with recent studies expanding on their implications for other areas of wellbeing and development. Bedtime routines encompass a wealth of different activities ranging from oral health hygiene practices (tooth brushing) to diet and social behaviours. Due to their multi-faceted nature, bedtime routines are an area of interest that spans numerous scientific and disciplinary boundaries. Bedtime routines include elements which are relevant to psychology, education, public health, dentistry, developmental studies and behavioural science. Due to links between activities undertaken as part of bedtime routines (i.e. tooth brushing, dietary habits, book reading etc.) and beneficial development and wellbeing outcomes, bedtime routines can be an area of interest for policy makers and health economists. In this early phase study, the inter-disciplinary nature of bedtime routines is reflected in the team’s expertise ranging from psychology to public health and dentistry. New insights into bedtime routines can promote inter-disciplinary work through increased awareness of this recurrent, dynamic behaviour and its health, wellbeing and developmental consequences. Also, as this early phase study aims to develop a behaviour change intervention for bedtime routines in first time parents, valuable lessons can be learnt and communicated with regard to the examination, understanding and changing of human behaviour, especially one of a recurrent and dynamic nature and its relationship to health outcomes.

Apart from academic beneficiaries and due to the diverse range of outcomes associated with good bedtime routines, additional beneficiaries from this early phase study include (a) first-time parents and parents with young children in general; (b) academics and researchers in public health, dental public health, psychology, behaviour change, digital health interventions, health policy, education and child wellbeing; (c) NHS and non-NHS practitioners especially GPs, dentists and the wider oral health team, nurses, health visitors, and child health specialists; (d) charities, trusts, local-regional-national level organisations specialising in child development-wellbeing (e.g. Children’s society), specific bedtime routines activities (e.g. Book Trust for book reading before bed) or the health of public in general (e.g. Public Health England); and (e) education bodies, schools and practitioners (e.g. Education Endowment Foundation).

The majority of listed individuals, groups and organisations will benefit from the general awareness of the outcomes and the innovative methodology of this early phase study. First-time parents and parents in general can benefit from increased awareness of the importance of bedtime routines, the constituent parts of a good bedtime routine and the importance of establishing a good bedtime routine from an early age. This benefit can be immediate for participating first-time parents. Non-participating parents can be reached and will benefit either directly (through the communication plan) or indirectly through the overall increased awareness and sharing of information between the research team and the wider public. Academics, researchers, policy makers and organisations can benefit from this early phase study especially its novel and innovative methodology when tackling their own research projects focusing on either human behaviour or similar populations. Also, researchers and academics working with deprived and ethnically diverse samples can gain valuable information due to the inclusion of such populations in this early phase study. Should this early phase study show promising results then a larger and more comprehensive study may produce positive differences in bedtime routines in first-time parents with short and long-term benefits for their children, parents themselves as well as public finances and the health service. These benefits can emerge from possible lower incidents of dental problems in children (i.e. fewer dental caries, less need for treatment, fewer hospitalisations, fewer dental extractions etc.), better quality of sleep, higher school readiness and school attainment with fewer school dropouts later in life etc.

## Conclusion

This proposed early stage feasibility and effectiveness study for the development and initial test of a bedtime routine intervention for first-time parents has the potential to influence a dynamic set of behaviours relating to child wellbeing and development. Through this study, initial yet important evidence on the effectiveness of such intervention can be gathered. This can allow for future work with more advanced study designs (longitudinal and RCT) with broader and more complex populations.

## Supplementary information

**Additional file 1.** Qualitative interview schedule based on the Theoretical Domains Framework. List of questions that participants will be asked as part of WP 1.1 (qualitative work). Each question covers at least one domain under TDF.

**Additional file 2.** Pre-Post Bedtime Routine Questionnaire. List of questions that will need to be adapted into a text survey for the parents to complete pre and post intervention regarding their bedtime routines.

**Additional file 3.** Pre-Post Child Sleep Habit Questionnaire. Standardised questionnaire to be used pre and post intervention to assess children’s quality of sleep.

**Additional file 4:.** Focus group guide. List of questions that the parents will be asked during their participation in the focus groups at the end of the study.

## Data Availability

The datasets used and/or analysed during the current study will be available from the corresponding author on reasonable request at the end of the study.
