# Supplementary material for: Bedtime Routines Intervention for Children (BRIC) using an automated text messaging system for behaviour change: study protocol for an early phase study
Source: Pilot Feasibility Stud. 2020 Feb 6;6:14. doi: 10.1186/s40814-020-0562-y (PMC7003486; doi:10.1186/s40814-020-0562-y)
Supplement: Supplementary file 1 — Additional file 1. Qualitative interview schedule based on the Theoretical Domains Framework. List of questions that participants will be asked as part of WP 1.1 (qualitative work). Each question covers at least one domain under TDF. [file 40814_2020_562_MOESM1_ESM.docx]

**Additional file A. Interview schedule based on the Theoretical Domains Framework**

**Interview schedule**

**0. Introduction**

0.1. Briefly discuss the scope of this interview as an additional source of information in trying to get a better understanding of bedtime routines in families with young children. Discuss overall set up, including time, audio recordings etc.

**1. Bedtime routines overview**

1.1. Can you describe your typical bedtime routine? What time does it start and end? What does it involve? In what order? Who’s involved?

**2. General knowledge & skills**

2.1. (KNOWLEDGE) How do you think a good bedtime routine looks like? What would be involved?

2.2. (KNOWLEDGE) You’ve mentioned X, Y and Z as things that would be involved in a ‘good’ bedtime routine – why do you think they’re important / why do they matter?

2.3. (KNOWLEDGE) Are you aware of guidelines/recommendations relating to bedtime routines? If yes, what do they usually include? If yes, who/how did you come across those guidelines/recommendations? (***Hint***: midwives, health visitors, internet etc.)

Following reply to Qs.1 and 2.1/2.2/2.3, provide a quick definition of what an optimal bedtime routine should include and use a visual aid to quickly refer to with regards to all 4 main components (tooth brushing, book reading, diet & use of electronics).

2.4 (SKILLS) What skills do you think you would need in order to be able to do things involved in a good bedtime routine? (point to prompt card)

2.5. (SKILLS) Which of these skills do you think you have? Are there ones you could do with improving?

2.6. (SOCIAL IDENTITY) Who is responsible for bedtime routines in your view? (***Hint***: me as parent, parents or others?)

2.7. (SOCIAL IDENTITY) Do you think other parents have good bedtime routines? Are they like you?

2.8. (SOCIAL INFLUENCES) (If someone’s involved) How do you feel about your partner’s/husband’s/wife’s etc. involvement in your bedtime routines? Do they help or hinder your activities? (If no one’s involved) Do you wish there was someone to help you with your bedtime routines? In what way someone else could be helpful for you during your bedtime routines?

2.9 (SOCIAL INFLUENCES) What do your friends / family think about your bedtime routine? Do you care what they think?

2.10 (SOCIAL INFLUENCES) What do your kids think about the different bits of this good bedtime routine (***Hint***: prompt card)? How important is it to you what they think about it?

**3. Current situation**

3.1. (BELIEFS CAPABILITIES) How easy or difficult is it for you to do your bedtime routine every night? Can you manage even when things are difficult?

3.2. (BELIEFS CAPABILITIES) How confident are you in completing your bedtime routine every night? (If confidence low) What would make you feel more confident? (If confidence high) What gives you confidence?

3.3. (EMOTIONS) How do you feel when you do manage to do all the things involved in your bedtime routine? What about when you don’t? What words describe how you typically feel during your bedtime routines (i.e. stressed, calm, happy, sad)?

3.4 (EMOTIONS) How does the way you feel as it’s coming up to bedtime influence whether or not you do the things involved in a good routine?

3.5. (MEMORY, ATTENTION & DECISION PROCESS) Are these different things (prompt card) things you do routinely, without thinking? Do they happen at a set time and in the same way every night or do you actively have to remember to do them?

3.6. (MEMORY, ATTENTION & DECISION PROCESS) Do your routines change between school days and weekends? If so, how are they different? Do you still complete all of your activities?

3.7. (MEMORY, ATTENTION & DECISION PROCESS) When you’re tired or have a lot on your mind, do you try and complete all activities? If not, how do you decide which activities to leave out?

3.8. (ENVIROMENT-RESOURCES) (If employed) Do you believe that your work affects your bedtime routines? If yes, in what way?

3.9. (ENVIROMENT-RESOURCES) Are there things about your home (e.g. where the bedrooms are, need to share rooms, noise, etc.) that make your bedtime routines easier or harder?

3.10. (ENVIROMENT-RESOURCES) Do you have access to the things you need to do the 4 things outlined here (prompt card), e.g. selection of books, toothbrushes, water/milk etc? If not, in what way?

3.11. (REINFORCEMENT) When you have a good bedtime routine, what happens and how do you feel straight afterwards? What were the immediate benefits?

3.12. (REINFORCEMENT) When you have a bad bedtime routine, what happens and how do you feel straight afterwards? What were the immediate consequences?

3.13. (GOALS) What is/are your end goal(s) when starting your bedtime routines on a nightly basis?

3.14. (GOALS) Compared to other things you need / want to get done, how important is it that you do all the things listed as part of your bedtime routine?

3.15. (BEHAVIOUR REGULATION) Do you monitor your bedtime routines in any way? If yes, how do you do it? If not, do you believe that it might be useful to monitor them?

3.16. (BEHAVIOUR REGULATION) (If monitor bedtime routines) What do you do when you notice that your bedtime routines are not as good as they used to be? Do you take any actions? If yes, what?

**4. Looking ahead**

4.1. (BELIEFS CONSEQUENCES) Looking ahead in the future, what do you think will happen if you have a good bedtime routine in place? For you, your child, your family or in general? What will happen if you don’t? Do some of these things (prompt card) matter more than other for the future?

4.2. (BELIEFS CONSEQUENCES) Do the future benefits outweigh the costs? How?

4.3. (OPTIMISM) Looking ahead, how do you feel about your upcoming bedtime routines for the days, weeks and years to come?

4.4. (OPTIMISM) Do you feel that regardless of what happens day to day, things will turn out fine in the end?

4.5. (INTENTIONS) Do you want to have a good bedtime routine? If yes, to what extent?

4.6 Do you feel ready and able to make any changes that are necessary to your existing bedtime routine?
