# Supplementary material for: Bedtime Routines Intervention for Children (BRIC) using an automated text messaging system for behaviour change: study protocol for an early phase study
Source: Pilot Feasibility Stud. 2020 Feb 6;6:14. doi: 10.1186/s40814-020-0562-y (PMC7003486; doi:10.1186/s40814-020-0562-y)
Supplement: Supplementary file 2 — Additional file 2. Pre-Post Bedtime Routine Questionnaire. List of questions that will need to be adapted into a text survey for the parents to complete pre and post intervention regarding their bedtime routines. [file 40814_2020_562_MOESM2_ESM.docx]

| **1** | How would you rate tonight's bed time routine from 0 (many problems, worst routine for a while) to 5 (best routine ever!). Please rate from 0 to 5. | 0 – Worst routine for a while  1- A lot of problems  2- A few problems  3 – Neither good or bad  4 – Good routine  5 – Best routine ever! |
| --- | --- | --- |
| **2** | What time did your child go to bed? If he/she is still awake, please reply "Awake" or "Not yet". | Write time child went to bed  Or Awake |
| **3** | Who was involved in tonight's routine? Mum, Dad, Both parents or someone else? Please reply "Mum", "Dad", "Both" or "Other". | Mum  Dad  Both  Other |
| **4** | Did your child eat or drink anything the hour (i.e. not his/her dinner) before bed? Please reply “Yes” or “No”. | Yes  No |
| **4.1** | What was it? Please briefly describe what your child ate and/or drank the hour (i.e. not dinner) before bed (i.e. glass of milk, chocolate, fruit etc.) | Write what the child ate before bed incl. drinks |
| **5** | Were your child’s teeth brushed tonight? Reply “Yes” or “No” | Yes  No |
| **5.1** | Who brushed them? Please reply "Child" or "Parent", or if you helped your child brush their teeth then reply "Together" | Child  Together  Parent |
| **6** | Did your child play video games, watch TV or use any electronic devices (incl. mobile phones, tablets etc.) the hour before bed? Please reply "Yes" or "No" | Yes  No |
| **6.1** | If Yes, can you specify what type of device? | TV  Computer  Table  Gaming console  Mobile phone  eBook reader |
| **7** | Did you read a story to your child before bed? Please reply "Yes" or "No" | Yes  No |

**Additional file B.1. Pre/Post Bedtime Routines Questionnaire (to be adapted**

**into text survey format)**
