# Supplementary material for: Bedtime Routines Intervention for Children (BRIC) using an automated text messaging system for behaviour change: study protocol for an early phase study
Source: Pilot Feasibility Stud. 2020 Feb 6;6:14. doi: 10.1186/s40814-020-0562-y (PMC7003486; doi:10.1186/s40814-020-0562-y)
Supplement: Supplementary file 3 — Additional file 3. Pre-Post Child Sleep Habit Questionnaire. Standardised questionnaire to be used pre and post intervention to assess children’s quality of sleep. [file 40814_2020_562_MOESM3_ESM.pdf]

CHILDREN'S SLEEP HABITS QUESTIONNAIRE (ABBREVIATED)

The following statements are about your child's sleep habits and possible difficulties with sleep. Think about the past week in your life when you answer the questions. If last week was unusual for a specific reason, choose the most recent typical week. Unless noted, check Always if something occurs every night, Usually if it occurs 5 or 6 times a week, Sometimes if it occurs 2 to 4 times a week, Rarely if it occurs once a week, and Never if it occurs less than once a week.

BEDTIME

Write in your child's usual bedtime: Weeknights _____:_____ am/pm

Weekends _____:_____ am/pm

	7 Always	5-6 Usually	2-4 Sometimes	1 Rarely	0 Never
1. Child goes to bed at the same time at night.	( )	( )	( )	( )	( )
2. Child falls asleep within 20 minutes after going to bed.	( )	( )	( )	( )	( )
3. Child falls asleep alone in own bed.	( )	( )	( )	( )	( )
4. Child falls asleep in parent's or sibling's bed.	( )	( )	( )	( )	( )
5. Child falls asleep with rocking or rhythmic movements.	( )	( )	( )	( )	( )
6. Child needs special object to fall asleep (doll, special blanket, stuffed animal, etc.).	( )	( )	( )	( )	( )
7. Child needs parent in the room to fall asleep.	( )	( )	( )	( )	( )
8. Child resists going to bed at bedtime.	( )	( )	( )	( )	( )
9. Child is afraid of sleeping in the dark.	( )	( )	( )	( )	( )

SLEEP BEHAVIOR

Write in your child's usual amount of sleep each day (combining nighttime sleep and naps): _____ hours and _____ minutes

	7 Always	5-6 Usually	2-4 Sometimes	1 Rarely	0 Never
10. Child sleeps about the same amount each day.	( )	( )	( )	( )	( )
11. Child is restless and moves a lot during sleep.	( )	( )	( )	( )	( )

	7<br>Always	5-6<br>Usually	2-4<br>Sometimes	1<br>Rarely	0<br>Never
12. Child moves to someone else's bed during the night (parent, sibling, etc.).	( )	( )	( )	( )	( )
13. Child grinds teeth during sleep (your dentist may have told you this).	( )	( )	( )	( )	( )
14. Child snores loudly.	( )	( )	( )	( )	( )
15. Child awakens during the night and is sweating, screaming, and inconsolable.	( )	( )	( )	( )	( )
16. Child naps during the day.	( )	( )	( )	( )	( )
Write in the number of minutes the nap usually lasts: _____ minutes					

WAKING DURING THE NIGHT

	7<br>Always	5-6<br>Usually	2-4<br>Sometimes	1<br>Rarely	0<br>Never
17. Child wakes up once during the night.	( )	( )	( )	( )	( )
18. Child wakes up more than once during the night.	( )	( )	( )	( )	( )

MORNING WAKE UP

Write in the time child usually wakes up in the morning: Weekdays _____:_____ am/pm

Weekends _____:_____ am/pm

	7<br>Always	5-6<br>Usually	2-4<br>Sometimes	1<br>Rarely	0<br>Never
19. Child wakes up by him/herself.	( )	( )	( )	( )	( )
20. Child wakes up very early in the morning (or, earlier than necessary or desired).	( )	( )	( )	( )	( )
21. Child seems tired during the daytime.	( )	( )	( )	( )	( )
22. Child falls asleep while involved in activities.	( )	( )	( )	( )	( )
