# Supplementary material for: Bedtime Routines Intervention for Children (BRIC) using an automated text messaging system for behaviour change: study protocol for an early phase study
Source: Pilot Feasibility Stud. 2020 Feb 6;6:14. doi: 10.1186/s40814-020-0562-y (PMC7003486; doi:10.1186/s40814-020-0562-y)
Supplement: Supplementary file 4 — Additional file 4:. Focus group guide. List of questions that the parents will be asked during their participation in the focus groups at the end of the study. [file 40814_2020_562_MOESM4_ESM.docx]

**Additional file C. Post intervention focus group schedule**

**A. Introduction (15 minutes)**

A.1. Moderator introduce himself.

A.2. Thank participants for agreeing to take part & provide brief overview of the structure for the group including information around timings, housekeeping, breaks and facilities.

A.3. *“Before the start of the group mention the following:”*

A.3.1. *“There are no right or wrong answers, but rather differing points of view. Please feel free to share your point of view even it differs from what others have said. Sometimes somebody in the group will have a strong opinion about something and you may have the exact opposite opinion. We would like to hear whatever you have to say.”*

A.3.2. *“Keep in mind that we are just as interested in negative comments as positive comments, and sometimes the negative comments are the most helpful.”*

A.3.3. *“I would like to record our conversation. The recording will be typed out, but everything you say will be anonymous. Your name and any names or places you mention will be taken out, so that if someone read it, they would not know who you are. We ask that you do not repeat anything you hear in this room.”*

A.3.4. *“To make it easier when we listen to the tape we’d like to ask you to speak up and try not to speak over one another, state your name before you speak so that when we listen to the tape, we can tell who is saying what. This can be a little difficult to get used to, so I’ll remind you as we go through.”*

A.3.5. *“My role here is to ask questions and listen, but I won’t be participating in the discussion. Feel free to talk with one another. There is a tendency in these discussions for some people to talk a lot and some people not to say much. But it is important for us to hear from each of you today because you have different experiences. So, if one of you is sharing a lot, I may ask you to let others talk. And if you aren’t saying much, I may ask for your opinion.”*

A.3.6. *“If at any time during the group you do not wish to answer a question that’s okay. If, at any stage, you wish to stop the audio recorder, please let me know. Do you have any questions?”*

**B. General views on bedtime routines (10-15 minutes)**

B.1. Do you think that bedtime routines are important when it comes to child wellbeing and development? If yes, why? If not, why?

B.2. Which areas of child wellbeing and development do you believe are more easily influenced by bedtime routines? Is it sleep, dental hygiene, something else?

B.3. What do you think constitutes a good bedtime routine? Any examples of a good bedtime routine?

**C. Views on text messaging system/intervention**

C.1. What is your overall view of the intervention you just completed?

C.2. What did you think about the personalisation of the text messages (i.e. first names)? Did it make you feel better than a generic text message or did it annoy you in any way?

C..3. What did you think about the information that was provided to you during the intervention? Did you find it useful?

C.4. Did the text messages cause any issues with your bedtime routines? Did it make it harder to concentrate on what you were doing?

C.5. Were you close to opt-out from the study at any stage? If yes, why and what kept you going?

C.6. Did your partner/husband/wife participate in the intervention? If yes, in what way?

C.7. What is their view on the whole system?

C.8. What is your view on the cost of the intervention? Did you notice it on your monthly use or do you have unlimited text messages?

**D. Moving forward**

D.1. In the future what would you like to see changed in the intervention?

D.2. Which elements of the current intervention would you keep and which ones would you lose?

D.3. Would you like to see further personalisation? If yes, in what format?

D.4. Would you like to see gamification (i.e. quizzes, fun elements etc.) in any future version of the intervention?

D.5. Would you prefer the intervention to be free of charge in the future?

D.6. Would you like to see the intervention being send to both parents or just one?

D.7. Would you *like additional options for example to receive recommendations on reading material/books and the option to buy/receive such material?*

*D.8. In the future, would you like to see/receive information on how other parents are doing (i.e. how many had a good bedtime routine etc.)*

*D.9. Would you change the frequency and duration of the text messages? If yes, in what way, which format is best for first-time parents in your opinion?*

*D.10. if such an intervention existed, who would you like to be responsible for informing you about it, registering you into the system and be your point of contact? For example, your GP, a midwife, a nurse/health visitor, academic/research staff or someone else?*

**E. Summary & Closing remarks (10 minutes)**

E.1. (Moderator summarises the discussion with help from the co-moderators.) What, if anything, have I missed from that summary?

E.2. Thank participants
